# Chondrogenic induction of human osteoarthritic cartilage-derived mesenchymal stem cells activates mineralization and hypertrophic and osteogenic gene expression through a mechanomiR

**DOI:** 10.1186/s13075-019-1949-0

**Published:** 2019-07-08

**Authors:** Nan Hu, Yun Gao, Chathuraka T. Jayasuriya, Wenguang Liu, Heng Du, Jing Ding, Meng Feng, Qian Chen

**Affiliations:** 1 Department of Rheumatology, the First Affiliated Hospital of Xi’an Jiaotong University, Xi’an, 710061 Shaanxi China; 2 Department of Orthopaedics, Warren Alpert Medical School of Brown University/Rhode Island Hospital, Providence, RI 02903 USA; 3 Bone and Joint Research Center, the First Affiliated Hospital and Frontier Institute of Science and Technology, Xi’an Jiaotong University, Xi’an, 710061 China; 4 Department of Orthopaedics, the First Affiliated Hospital of Xi’an Jiaotong University, Xi’an, 710061 Shaanxi China

**Keywords:** miR-365, Mesenchymal stem cells, Osteoarthritis, OA stem cells

## Abstract

**Background:**

While bone marrow-derived mesenchymal stem cells (BMSC) are established sources for stem cell-based cartilage repair therapy, articular cartilage-derived mesenchymal stem cells from osteoarthritis patients (OA-MSC) are new and potentially attractive candidates. We compared OA-MSC and BMSC in chondrogenic potentials, gene expression, and regulation by miR-365, a mechanical-responsive microRNA in cartilage (Guan et al., FASEB J 25: 4457–4466, 2011).

**Methods:**

To overcome the limited number of OA-MSC, a newly established human OA-MSC cell line (Jayasuriya et al., Sci Rep 8: 7044, 2018) was utilized for analysis and comparison to BMSC. Chondrogenesis was induced by the chondrogenic medium in monolayer cell culture. After chondrogenic induction, chondrogenesis and mineralization were assessed by Alcian blue and Alizarin red staining respectively. MiRNA and mRNA levels were quantified by real-time PCR while protein levels were determined by western blot analysis at different time points. Immunohistochemistry was performed with cartilage-specific miR-365 over-expression transgenic mice.

**Results:**

Upon chondrogenic induction, OA-MSC underwent rapid chondrogenesis in comparison to BMSC as shown by Alcian blue staining and activation of ACAN and COL2A1 gene expression. Chondrogenic induction also activated mineralization and the expression of hypertrophic and osteogenic genes in OA-MSC while only hypertrophic genes were activated in BMSC. MiR-365 expression was activated by chondrogenic induction in both OA-MSC and BMSC. Transfection of miR-365 in OA-MSC induced chondrogenic, hypertrophic, and osteogenic genes expression while miR-365 inhibition suppressed the expression of these genes. Over-expression of miR-365 upregulated markers of OA-MSC and hypertrophy and increased OA scores in adult mouse articular cartilage.

**Conclusions:**

Induction of chondrogenesis can activate mineralization, hypertrophic, and osteogenic genes in OA-MSC. MiR-365 appears to be a master regulator of these differentiation processes in OA-MSC during OA pathogenesis. These findings have important implications for cartilage repair therapy using cartilage derived stem cells from OA patients.

**Electronic supplementary material:**

The online version of this article (10.1186/s13075-019-1949-0) contains supplementary material, which is available to authorized users.

## Background

Osteoarthritis (OA), the most prevalent degenerative arthropathy, is a common cause of disability due to articular cartilage degeneration and bone remodeling. For decades researchers have been trying to identify an abundant and suitable cell population with which to replenish injured or diseased hyaline cartilage. To date, several avenues of cell-based cartilage repair therapies have been explored [1]. Bone marrow-derived mesenchymal stem cells (BMSC) are one of the established stem cell sources for its capability of chondrogenic differentiation and formation of hyaline-like cartilage tissue [2].

Articular cartilage-derived mesenchymal stem cells from osteoarthritis patients (OA-MSC) are new and potentially attractive candidates for cell-based cartilage repair. They are readily available from surgery without immunological rejection for cell transplant [3]. However, they reside in OA cartilage of geriatric individuals, exhibiting different cell surface marker profile and differentiation ability than that of MSC derived from normal articular cartilage origin [3–6]. Thus, to utilize OA-MSC for cartilage repair, further characterization is needed regarding its capacity for chondrogenesis and the key molecules that regulate this process.

MicroRNAs (miRNAs) have profound effects on the cellular phenotype and biological function in various tissues [7]. Numerous studies have shown that miRNAs are important regulators of diverse biological processes, such as differentiation, development, and tumorigenesis [8–10]. MicroRNAs (miRNAs) are a class of endogenous noncoding RNAs, approximately 20–25 nucleotides in length, that regulate one third of all mammalian gene expression at the post-transcriptional level by targeting mRNAs via binding to complementary sequences in 3′ untranslated region (3′UTR) [11]. MiRNA-365 has been identified as a mechano-responsive miRNA (mechanomiR) that potently stimulates chondrocyte hypertrophy by transmitting mechanical as well as inflammatory signals [12, 13]. Over-expression of miRNA-365 in cartilage was correlated with early onset of OA by promoting hedgehog signaling and hypertrophic marker expression during aging [13]. Yet the role of miR-365 in OA-MSC remains unknown.

Since miR-365 was correlated with chondrocyte hypertrophy and OA pathogenesis, we hypothesize that miR-365 may also play a role in regulating OA-MSC differentiation during chondrogenesis. If so, it may be used to modulate OA-MSC differentiation during cell-based cartilage repair. In the present study, we compared chondrogenesis potentials as well as gene expression regulated by miR-365 between OA-MSC and BMSC and analyzed the role of miR-365 in regulating cartilage cells hypertrophy in vitro and in vivo.

## Methods

### Mesenchymal stem cells

Multiple cell lines of OA articular cartilage-derived mesenchymal stem cells (OA-MSC), normal articular cartilage-derived progenitor cell line 3 (nCPC), primary OA articular chondrocytes, and primary OASC were established after the use of discarded knee cartilage samples from patient surgery was approved by IRB as previously described [4]. Human BMSC cells and culture media were obtained commercially (ATCC, Manassas, VA USA). The study was performed using one of the OA-MSC lines OASC2, which has been characterized previously [3]. First, chondrogenesis of OASC2 was induced for 3, 7, and 14 days in vitro, followed by quantitative real-time reverse transcription polymerase chain reaction (qPCR), western blot, and histological staining (Alcian blue and Alizarin red), and second, OASC2 was transfected with miR-365 mimic or miR-365 inhibitor (antagomir) followed by gene expression analysis. BMSC were treated the same way as OASC2 for control.

### MSC culture

OASC2 and BMSC were cultured at 37 °C in 5% CO_2_ in their respective growth medium for 1 week. After the cells reached confluency, they were sub-cultured at 1.25 × 10^5^ cells/well in 12-well plates. For each group at each time point, cells were treated with StemPro® Chondrogenesis Differentiation kit (Thermo Scientific, Waltham, MA USA) to induce chondrogenic differentiation or with stem cell growth medium (DMEM, 1 mM glutamine, 100 mM sodium pyruvate, 100 mg/mL ascorbic acid) as control. Medium was changed every 4 days. On days 3, 7, and 14 of chondrogenic differentiation, outcome parameters were assessed by qPCR, western blot, and histological staining (Alcian blue staining and Alizarin red staining). Alcian blue- and Alizarin red-stained wells were scanned and analyzed using ImageJ software. OASC2 and BMSC were transfected with Lipofectamine 3000 (Thermo Scientific, Waltham, MA USA) in 12-well plates. Forty-eight hours after transfection, cells were lysed in QIAzol lysis reagent (Qiagen, Hilden, Germany) for RNA isolation and real-time qPCR analysis.

### Transgenic mice

MiR-365 flox transgenic founders of the C57/BL6 background were generated at Brown University Mouse Transgenic and Gene Targeting Facility as previously described [14]. Upregulation of miR-365 in cartilaginous tissues in vivo was achieved by crossing miR-365 fl +/− mice with Col2a1-Cre +/− mice. It resulted in more than six folds over-expression of miR-365 levels in cartilage from miR-365 fl +/−; Col2a1-Cre +/− mice (miR-365Tg) in comparison to its littermate control mice miR-365 fl −/−; Col2a1-Cre +/− (Cre-only) [14]. The miR-365 Tg mice develop normally except for a slightly shorter femur [14]. All mice were housed, handled, and euthanized in accordance with federal and institutional guidelines. Animal protocols were approved by Lifespan IACUC animal studies committee for this project.

### Transfection

Cells were seeded onto desired size plates to reach 70–90% confluence and transfected with miR-365 mimic (miRIDIAN™ miRmimic human has-miR-365a-3p, C-300666-03, 5 nmol, Dharmacon, Lafayette, CO, USA) or miRNA mimic negative control (miRIDIAN™, CN-002000-01-05, 5 nmol, Dharmacon, Lafayette, CO, USA) or miR-365 inhibitor (miRIDIAN™ miRinhibitor has-miR-365a-3p, IN-300666-05, 5 nmol, Dharmacon, Lafayette, CO, USA) or inhibitor negative control (miRIDIAN™, IN-001005-01-05, 5 nmol, Dharmacon, Lafayette, CO, USA). MiR-365 mimic, miRNA mimic negative control, miR-365 inhibitor, and inhibitor negative control were used at a final concentration of 25 nM unless otherwise stated. Lipofectamine 3000 (Invitrogen®, Waltham, MA, USA) was used as transfection reagents. Medium is changed 24 h after transfection. In 48 or 72 h post-transfection, cells were lysated in either QIAzol for RNA purification and real-time PCR analysis or ice-cold lysis buffer containing protease inhibitor and phosphatase inhibitor for western blot analysis.

### Quantitative real-time PCR

Total RNAs, including mRNAs and miRNAs, were isolated from undifferentiated and chondrogenic differentiated OASC2 and BMSC respectively using the miRNA isolation kit according to the manufacturer’s protocol (Qiagen, Hilden, Germany). RNA concentration and quality were determined using Nanodrop ND-1000 spectrophotometer (Thermo Scientific, Waltham, MA USA). Five hundred nanograms (ng) of RNA was reverse transcribed using the miScriptIIRT Kit (Qiagen, Hilden, Germany). Quantitative real-time PCR (qPCR) was performed with the SYBR Green PCR Master mix (Qiagen, Hilden, Germany) using the Bio-Rad CFX96 real-time PCR detection system (Bio-Rad, Hercules, CA USA). Sense and antisense primers were shown in Table 1. The primers for human (hsa) U6 and miR-365 were purchased from Qiagen miScript Primer Assay system and their sequences were not fully available online. All reactions were run in duplicate. Crossing point (Cp) values for miR-365 were normalized to U6 snRNA, while Cp values for mRNAs were normalized to 18S. Relative gene/miRNA expression was determined using the 2-∆∆Ct method.

**Table 1:** Primer list

Genes	Forward (5′–3′)	Reverse (5′–3′)
18S	CGGCTACCACATCCAAGGAA	GCTGGAATTACCGCGGCT
SOX9	CCCCAACAGATCGCCTACAG	GAGTTCTGGTCGGTGTAGTC
ACAN	AGTCCTCAAGCCTCCTGTACTCA	CGGGAAGTGGCGGTAACA
COL2A1	GCCTGGTGTCATGGGTTT	GTCCCTTCTCACCAGCTTTG
RUNX2	GGCAGGCACAGTCTTCCC	GGCCCAGTTCTGAAGCACC
IHH	CCGCGTGGCAGCTGTCTCTAC	CCCCATGCCAAGCTGTGAAAG
COL10A1	GCCCACAGGCATAAAAGGCCC	GAAGGACCTGGGTGCCCTCGA
ADAMTS5	GGCCGTGGTGAAGGTGGTGG	GCTGCGTGGAGGCCATCGTC
COL1A1	CAGGAGGCACGCGGAGTGTG	GGCAGGGCTCGGGTTTCCAC
OCN	AGGGCAGCGAGGTAGTGAAG	ATAGGCCTCCTGAAAGCCG

### Western blot

All pre-treated samples were washed with ice-cold PBS and lysated in lysis buffer (M-PER, Pierce, Illinois, IL USA) plus protease inhibitor phenylmethylsulfonyl fluoride (Halt, Pierce, Illinois, IL USA) for 30 min on ice with constant agitation. The lysates were centrifuged at 12,000×*g* for 15 min at 4 °C. The supernatants were collected, and the protein concentrations were determined using BCA assay (Pierce, Illinois, IL USA). Samples were heated for 5 min at 95 °C. Equal amount of proteins for each sample were separated by 10% SDS-polyacrylamide gel and then transferred to polyvinylidene difluoride (PVDF) membrane (Bio-Rad, Hercules, CA USA) for 70 min at 100 V. The membrane was blocked with 5% bovine serum albumin (BSA) in Tris-buffered saline-Tween 20 (0.1%) (TBS-T) for 1 h at room temperature, followed by incubation with primary antibodies at 4 °C overnight (Table 2). Membrane was rinsed with TBS-T for 10 min for a total of 5 times and incubated with goat anti-rabbit-Alexa Fluor 680 and donkey anti-mouse-Alexa Fluor 680 (Molecular Probes, Eugene, OR, USA) for 1 h at room temperature. The blots were scanned using an Odyssey fluorescence scanner (LI-COR Biosciences, Lincoln, NE, USA). The densitometry of each blot was analyzed by NIH ImageJ software according to instruction at ImageJ.net.

**Table 2:** Antibody list

Proteins	Molecular weight (KD)	Antibody origin	Vendor	Catalog#
β-ACTIN	45	Rabbit	Cell Signaling	cs-4970S
α-TUBULIN	50	Mouse	Abcam	ab-7291
SOX9	70	Rabbit	Abcam	ab-185966
RUNX2	57–60	Rabbit	Abcam	ab-23981
COL10A1	66	Mouse	Abcam	ab-49945
OCN	11	Rabbit	Abcam	ab-93876
CD166	IHC	Mouse	Abcam	ab-175428
MMP13	60/IHC	Rabbit	Abcam	ab-39012
Col10a1	IHC	Rabbit	Abcam	ab-58632
ADAMTS5	75	Rabbit	Santa Cruz	sc-83186

### Immunohistochemistry

Mouse knees from 6-month-old miR-365 Tg mice and age-matched control littermates were decalcified, fixed overnight in formalin solution and paraffin embedded. The blocks were then sectioned (3.0 μm thick), mounted onto slides, cleared with xylene, and rehydrated using sequential incubation in 100%, 95%, 70%, and 50% ethanol solution. Sample slides were rinsed in deionized water, and antigen retrieval was performed using sodium citrate buffer (10 mM sodium citrate, pH 6) and an 850 W microwave. Slides were blocked overnight at 4 °C using 1% bovine serum albumin in 1× PBS to eliminate non-specific binding of the primary antibody. Slides were stained with a monoclonal mouse antibody (diluted 1:100 in 1× PBS, 1% BSA) against CD166, Col2a1, and Mmp13 (Abcam, Cambridge, MA, USA), overnight at 4 °C. Sections were then stained for 30 min with a green fluorescently labeled anti-mouse secondary antibody Alexa Fluor ab150105 (Abcam, Cambridge, MA, USA). Fluorescent images were acquired at × 20 magnification using a Nikon Eclipse 90*i* Digital Imaging System. A total number of cells and CD166 positively stained cells in the cartilage were manually counted on sections from three different animals per group respectively. The percentage of Col10a1 and Mmp13 positively stained area was calculated by ImageJ.

### Safranin O histology

Paraffin sections were de-paraffinized in 2 changes of xylene, 10 min each, followed by re-hydration in 2 changes of 100% alcohol, 5 min each; 2 changes of 95% alcohol, 5 min each; and 70% alcohol for 5 min. Sections were then rinsed in running tap water for 2 min before stained with 0.4% fast green solution (Sigma, Cat. F-7258, St. Louis, MO 63103 USA) for 2 min; however, the latter timing must be empirically controlled to assure desired coloration. Stained sections were then quickly rinsed with 1% acetic acid solution (Sigma, Cat. 695092-500ML-GL, St. Louis, MO 63103 USA) for no more than 10–15 s. 0.1% Safranin O Solution was used for staining proteoglycan; however, the actual timing must be carefully determined based on actual coloring condition, for maximally 10 min. After Safranin O staining, sections were submerged in 2 changes of 95% alcohol, 2 min each, 2 changes of 100% alcohol, 2 min each for de-hydration. Lastly, sections were cleared in 2 changes of xylene, 2 min each, and mounted using resinous mounting medium (ACRYMOUNT™, Cat. SL80-4, McKinney, TX 75069 USA). To histologically evaluate OA severity, we quantified Safranin O-stained knee sections according to OARSI semi-quantitative system as previously described [15]. Evaluators were blinded to the samples.

### Statistical analysis

GraphPad Prism 6 Software was used to perform statistical analyses. The Student’s unpaired *t* test was applied to determine the differences between the gene expression levels, which represent mean values ± SD (error bars). Gene expression data are expressed as fold differences—chondrogenic differentiated MSCs compared with undifferentiated MSCs. Significance was determined using raw data and assigned at *p* value < 0.05. There is a minimum of *n* = 3 for all groups.

## Results

### Chondrogenic induction of OA-MSC but not BMSC in monolayer cell culture

**Fig. 1:**
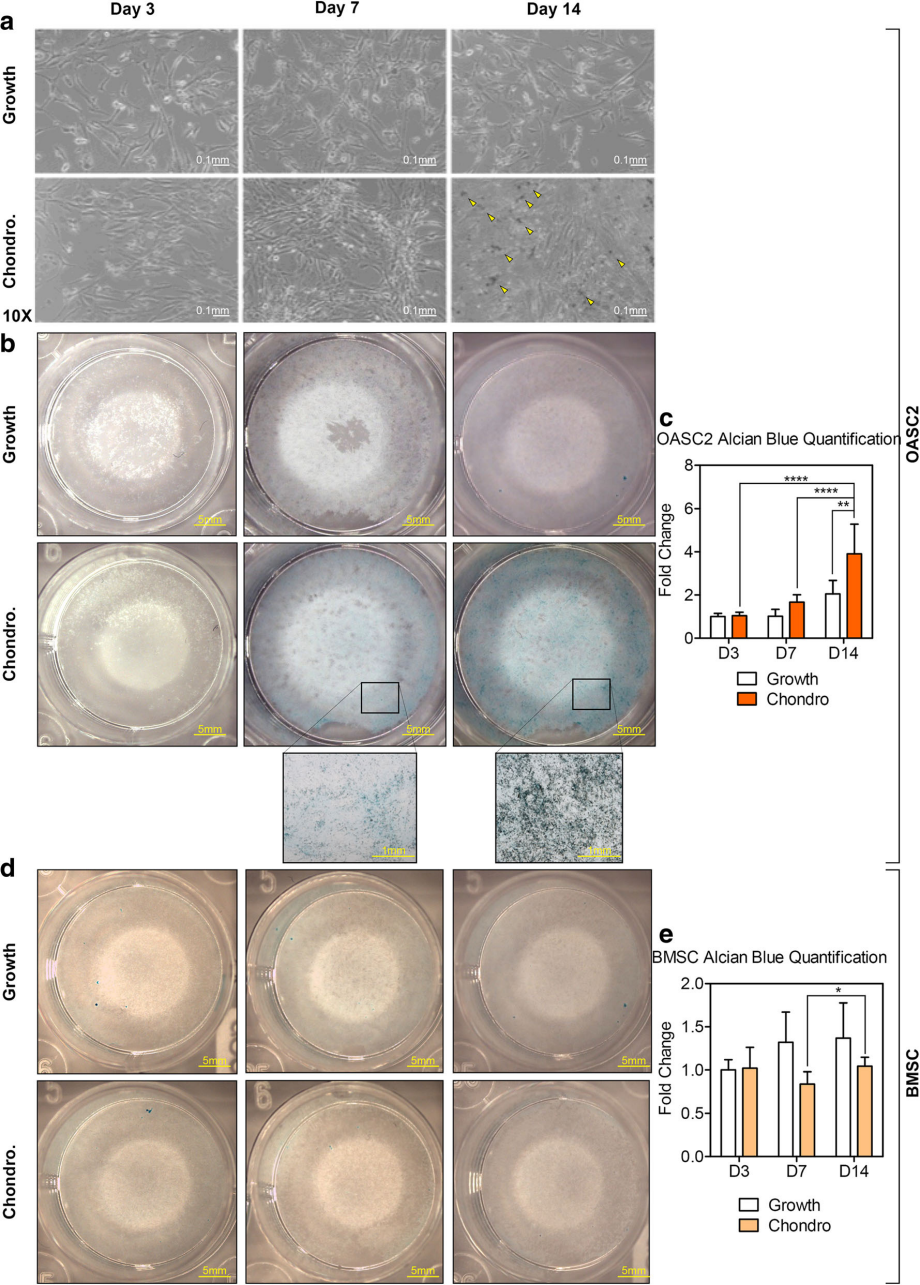
Chondrogenic induction of OASC2 and BMSC. **a** Comparison of changes in the morphology of OASC2 in chondrogenesis medium (Chondro.) with control (Growth) medium. In chondrogenesis medium, the cells gradually increased in numbers, volume and formed mineral deposition (yellow arrows) by day 14. **b** Alcian blue staining showed proteoglycans (blue) in OASC2 in chondrogenesis medium (Chondro.) from day 7. **c** Quantification of Alcian blue staining in OASC2 calculated by ImageJ software. **d** Alcian blue staining of BMSC in basal medium (Growth) or in chondrogenesis medium (Chondro.). **e** Quantification of Alcian blue staining in BMSC calculated by ImageJ software. Data are presented as mean ± SD from at least 3 sample images for each group; *p ≤ 0.05; **p ≤ 0.01; ****p ≤ 0.0001, relative to indicated control groups respectively

The cellular morphology of OASC2 presented a long tubular structure, which was similar to that of BMSC before chondrogenic differentiation (Fig. 1a). Upon induction of chondrogenesis, human OASC2 gradually increased in cell number and volume with more extracellular matrix deposition (Fig. 1a, Chondro.). OASC2 was stained with Alcian blue to determine the presence of glycosaminoglycans. Alcian blue staining was visible as early as day 7 under chondrogenesis conditions and progressively increased in the accumulated matrix at day 14 (Fig. 1b, c). In contrast, there was no visible Alcian blue staining in the BMSC group by day 14 under chondrogenesis conditions (Fig. 1d, Chondro.). This was consistent with the previous observation that 3D pellet culture was required for inducing chondrogenesis in BMSC [16]. Thus, upon chondrogenic induction in monolayer cell culture, OASC2 underwent rapid chondrogenesis in comparison to BMSC (Additional files 1 and 2).

**Fig. 2:**
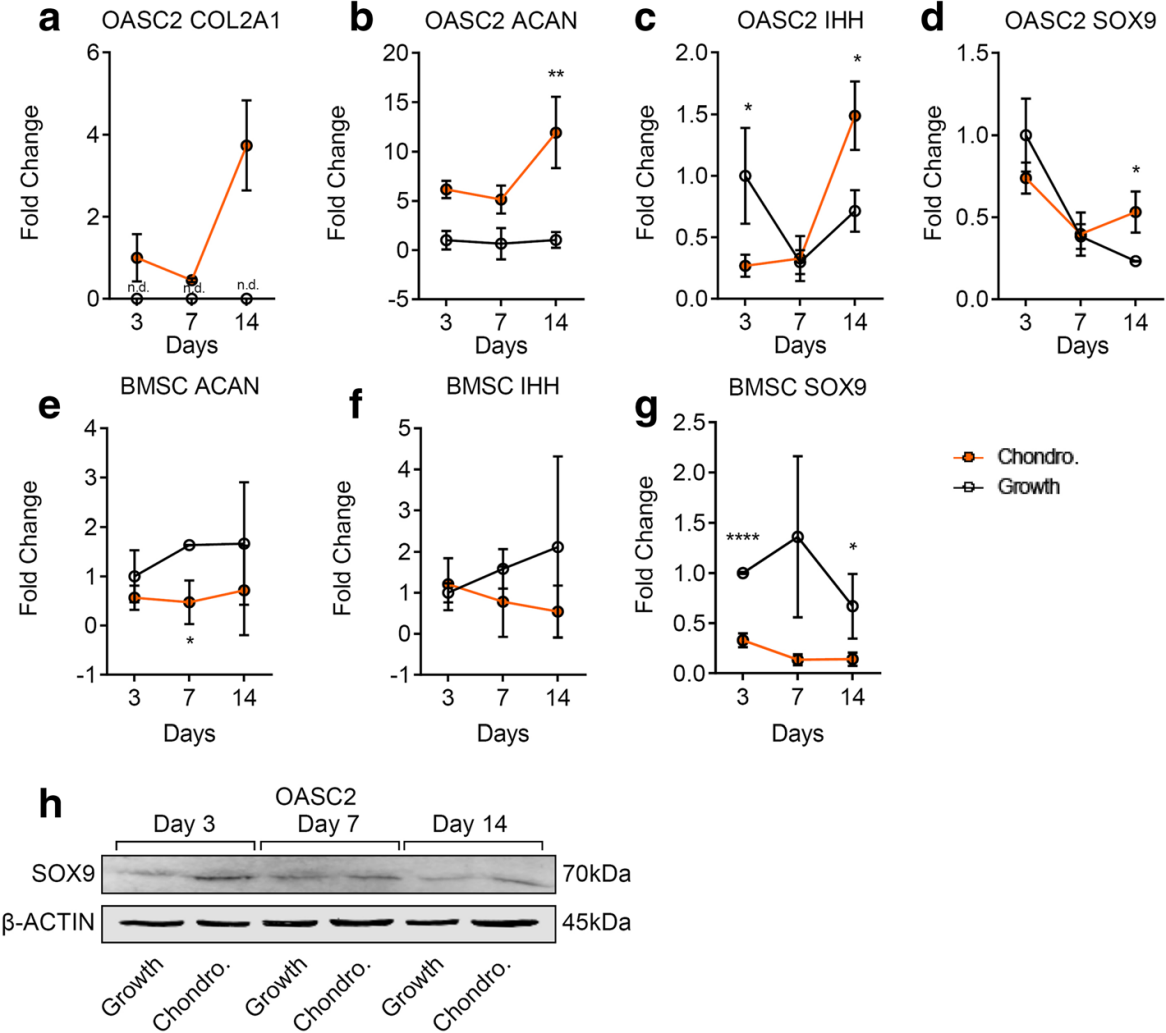
Gene expression of chondrogenic markers in OASC2 and BMSC during chondrogenic induction. **a** A dot graph showing quantification of COL2A1 mRNA level in the chondrogenic induction group (Chondro.) and control group (Growth) in OASC2. The expression levels of COL2A1 were significantly increased under chondrogenic condition than control; n.d., not detected. **b** Quantification of ACAN mRNA level in the chondrogenic induction group and control group in OASC2. The expression levels of ACAN were significantly increased under chondrogenic condition than control. **c** Quantification of IHH mRNA level in the chondrogenic induction group and control group in OASC2. The expression levels of IHH increased significantly since day 7 after induction. **d** Quantification of SOX9 mRNA level in the chondrogenic induction group and control group in OASC2. The expression levels of SOX9 increased significantly since day 7 after induction. **e** Quantification of ACAN mRNA level in the chondrogenic induction group and control group in BMSC. The ACAN levels were lower after chondrogenic induction. **f** Quantification of IHH mRNA level in the chondrogenic induction group and control group in BMSC. The IHH levels were lower after chondrogenic induction than control. **g** Quantification of SOX9 mRNA level in the chondrogenic induction group and control group in BMSC. SOX9 levels were suppressed significantly after chondrogenic induction. **h** Western Blot analysis showing SOX9 protein expression after chondrogenic induction in OASC2. The SOX9 protein levels were increased after chondrogenic induction. Quantification of western blot is presented in Additional file 1: Figure S1a. Data are presented as mean ± SD from at least 3 sample images for each group; *p ≤ 0.05; **p ≤ 0.01; ****p ≤ 0.0001, relative to indicated control groups respectively

Upon chondrogenic induction, the mRNA levels of chondrogenic markers COL2A1 (Fig. 2a) and ACAN (Fig. 2b) were increased during OASC2 differentiation, while the expression levels of IHH (Fig. 2c) and SOX9 (Fig. 2d) were increased after day 7. Western blot analysis indicated the increase of SOX9 protein levels by chondrogenic induction (Fig. 2h). In comparison, chondrogenic markers ACAN (Fig. 2e) and SOX9 (Fig. 2g) were decreased in BMSC upon chondrogenic induction in monolayer cell culture, consistent with the lack of Alcian blue staining (Fig. 1d, e).

### Chondrogenic induction activated mineralization and hypertrophic and osteogenic gene expression in OA-MSC

**Fig. 3:**
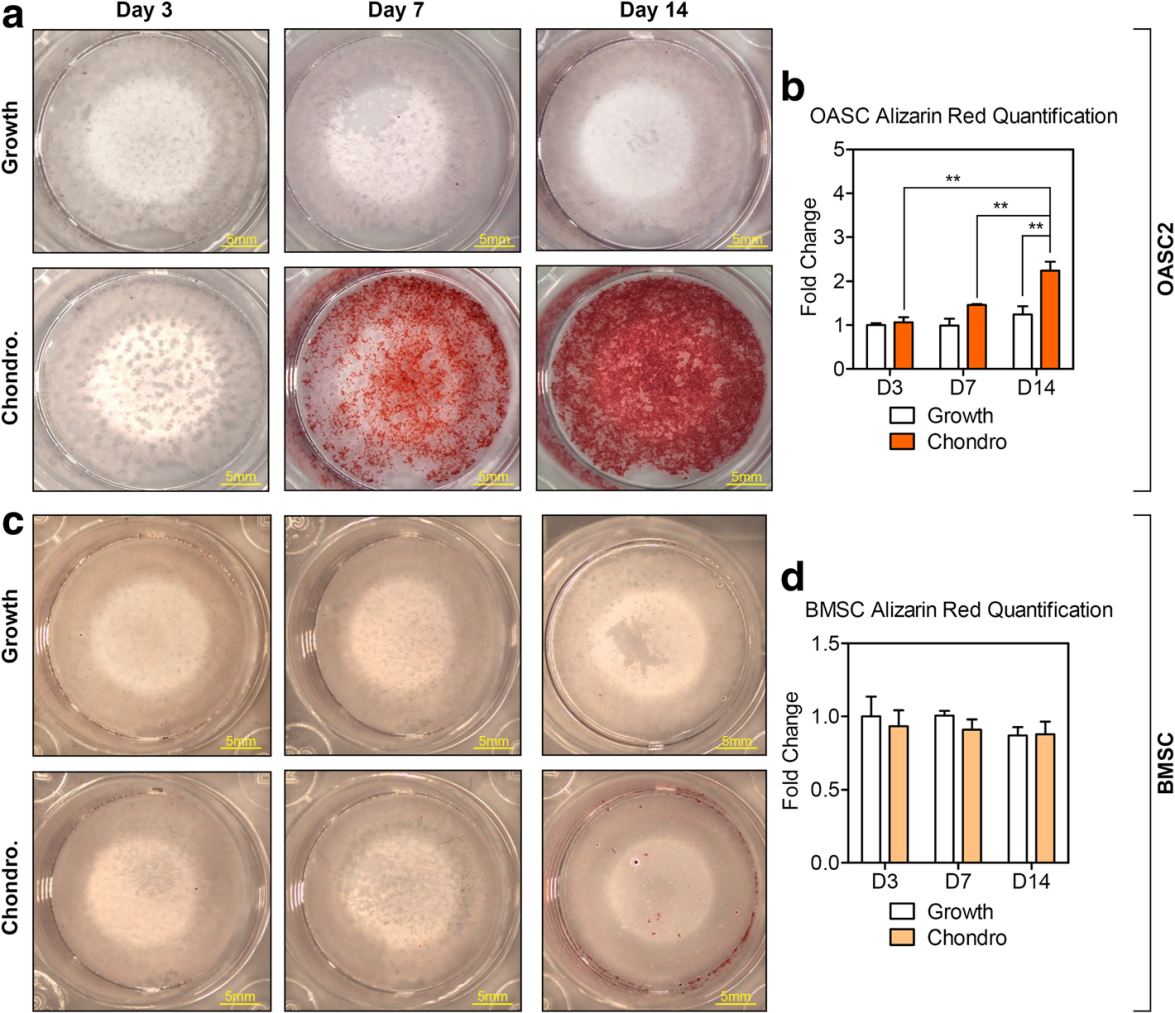
Comparison of Alizarin-Red Staining between OASC2 and BMSC in growth medium and chondrogenic induction medium. **a** In OASC2, Alizarin Red Staining was visible in chondrogenesis medium (Chondro.) as early as day 7 and presented a progressive increase in the calcium content in the accumulated matrix by day 14. **b** Quantification of Alizarin Red staining in OASC2 by ImageJ software. **c** Minimal Alizarin Red staining in BMSC when cultured in basal medium (Growth) or chondrogenesis medium (Chondro.) by day 14. **d** Quantification of Alizarin red staining in BMSC by ImageJ software. Data are presented as mean ± SD from at least 3 sample images for each group. **: *p* ≤ 0.01, relative to indicated control groups respectively

We noticed the deposition of minerals in OASC2 by day 14 after chondrogenic induction under a phase contrast microscope **(**Fig. 1a, D14 Chondro.). To determine whether mineralization was activated by chondrogenic induction in OASC2, we performed Alizarin red staining. In OASC2, Alizarin red stains were greatly increased at day 7 after chondrogenic induction and reached a peak at D14 (Fig. 3a, b). In contrast, only residual Alizarin red stains were noticeable at D14 after chondrogenic induction in BMSC (Fig. 3b, d).

**Fig. 4:**
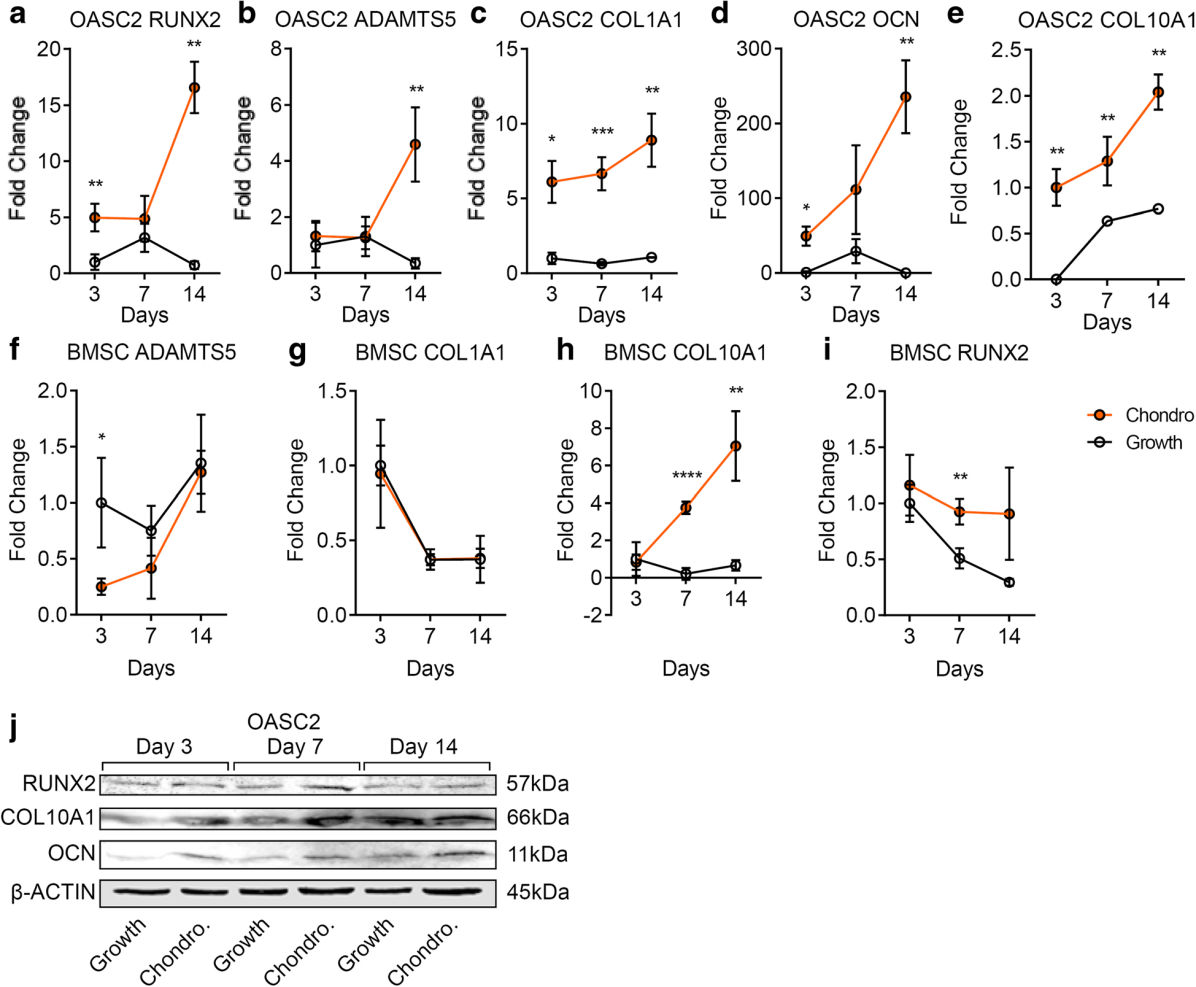
Gene expression of hypertrophic and osteogenic markers in OASC2 and BMSC during chondrogenic induction. **a** A dot graph showing quantification of RUNX2 mRNA level in the chondrogenic induction group (Chondro.) and control group (Growth) in OASC2. The expression levels of RUNX2 were significantly by chondrogenic induction at days 3 and 14. **b** Quantification of ADAMTS5 mRNA level in the chondrogenic induction group and control group in OASC2. The expression levels of ADAMTS5 were significantly higher than control on day 14. **c** Quantification of COL1A1 mRNA level in the chondrogenic induction group and control group in OASC2. The expression levels of COL1A1 were significantly higher under chondrogenic induction than control. **d** Quantification of OCN mRNA levels in the chondrogenic induction group and control group in OASC2. OCN levels were significantly higher under chondrogenic induction than control. **e** Quantification of COL10A1 mRNA level in the chondrogenic induction group and control group in OASC2. The COL10A1 expression levels were significantly higher under chondrogenic induction than control. **f** Quantification of ADAMTS5 mRNA level in the chondrogenic induction group and control group in BMSC. The ADAMTS5 expression level was significantly lower at day 3 after chondrogenic induction. **g** Quantification of COL1A1 mRNA levels in the chondrogenic induction group and control group in BMSC. There was no significant difference of COL1A1 expression levels in response to chondrogenic induction. **h** Quantification of COL10A1 mRNA level in the chondrogenic induction group and control group in BMSC. There was a significant increase of COL10A1 expression level at days 7 and 14 after chondrogenic induction. **i** Quantification of RUNX2 mRNA level in the chondrogenic induction group and control group in BMSC. Chondrogenic treatment significantly increased RUNX2 expression at day 7 after induction. **j** Western blot analysis showing RUNX2, COL10A1, and OCN protein expression during chondrogenic induction in OASC2. RUNX2, The COL10A1, and OCN protein levels were increased by chondrogenic induction in OASC2. Quantification of western blot is presented in Additional file 1: Figure S1b, c, d. Data are presented as mean ± SD from at least 3 sample images for each group; *p ≤ 0.05; **p ≤ 0.01; ***p ≤ 0.001; ****p ≤ 0.0001, relative to indicated control groups respectively

Mineralization is associated with chondrocyte hypertrophy and osteogenesis. To determine whether the expression of hypertrophic and osteogenic genes were induced by chondrogenic induction, we quantified the markers including COL10A1, RUNX2, ADAMTS5, COL1A1, and OCN. All the markers were increased significantly by chondrogenic induction in the OASC2 group (Fig. 4a–e). In contrast, in the BMSC group, only chondrocyte hypertrophic markers COL10A1 and RUNX2 were increased by chondrogenic induction, but COL1A1 and ADAMTS5 were not (Fig. 4f–i). Western blot analysis indicated that the protein levels of RUNX2, COL10, and OCN were increased by chondrogenic induction in OASC2 (Fig. 4j).

### MiR-365 induces chondrogenic, hypertrophic, and osteogenic gene expression in OA-MSC

**Fig. 5:**
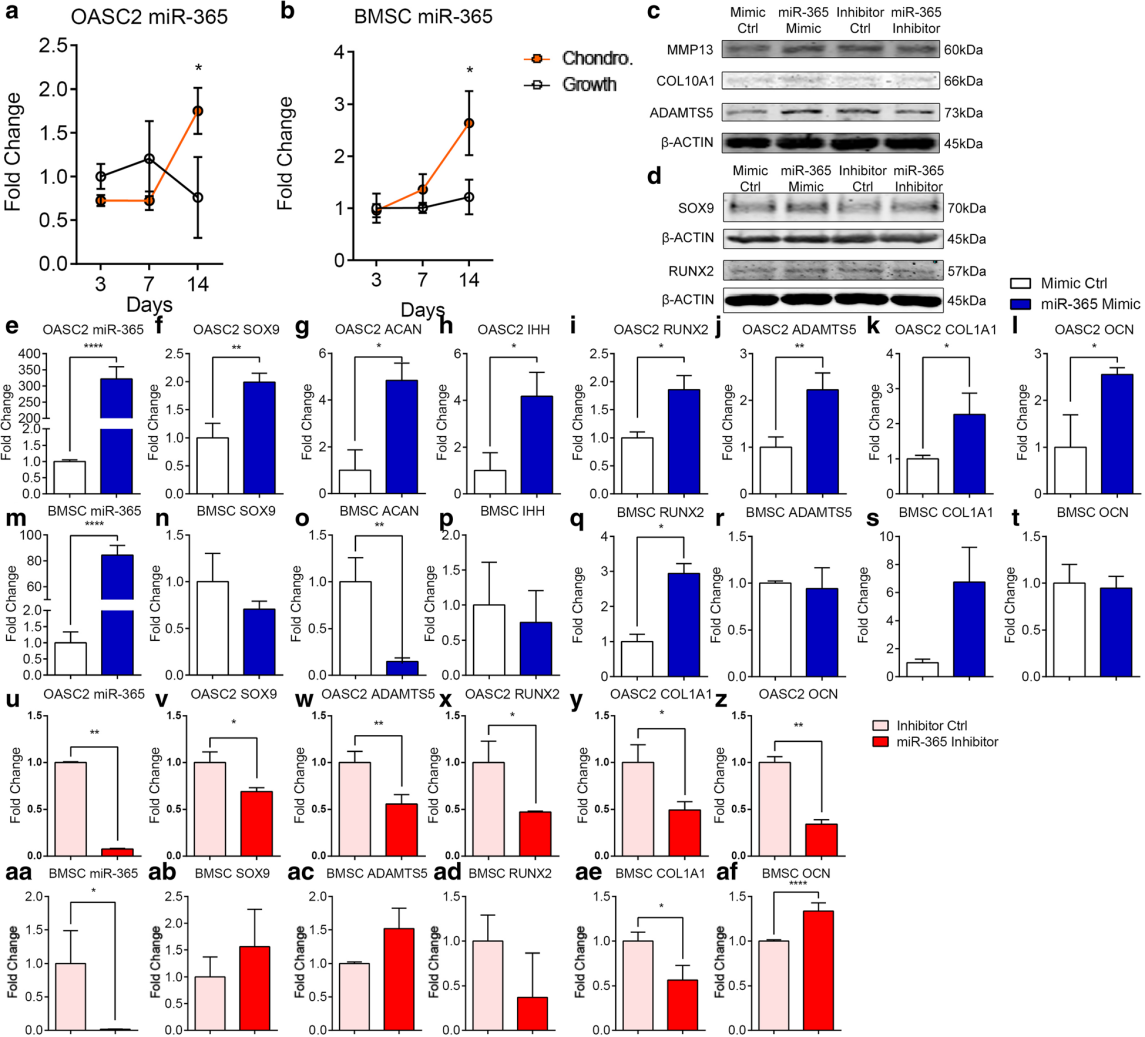
The chondrogenic, hypertrophic, and osteogenic markers expression in undifferentiated OASC2 and BMSC upon transfection of miR-365 or its inhibitor. **a** A dot graph showing quantification of miR-365 RNA level in the chondrogenic induction group (Chondro.) and control group (Growth) in OASC2. The expression level of miR-365 was significantly increased by chondrogenic induction at Day 14 in OASC2. **b** MiR-365 RNA levels in BMSC. The expression level of miR-365 was significantly increased by chondrogenic induction at Day 14 in BMSC. **c**, **d** The protein expressions of MMP13, COL10A1, ADAMTS5, SOX9, RUNX2, and β-ACTIN in OASC2 transfected with miR-365 mimic and miR-365 inhibitor; western blot images are representative of three samples (*n* = 3). Quantification of western blot is presented in Additional file 1: Figure S2. **e** MiR-365 levels were significantly increased after transfection of miR-365 (miR-365 mimic) in comparison to transfection control (Mimic Ctrl) in OASC2. **f** The SOX9 mRNA level was significantly increased by miR-365 transfection in OASC2. **g** The ACAN mRNA level was significantly increased by miR-365 transfection in OASC2. **h** The IHH mRNA level was significantly increased by miR-365 transfection in OASC2. **i** The RUNX2 mRNA level was significantly increased by miR-365 transfection in OASC2. **j** The ADAMTS5 mRNA level was significantly increased by miR-365 transfection in OASC2. **k** The COL1A1 mRNA level was significantly increased by miR-365 transfection in OASC2. **l** The OCN mRNA level was significantly increased by miR-365 transfection in OASC2. **m** MiR-365 levels were significantly increased after transfection of miR-365 (miR-365 mimic) in comparison to transfection control (Mimic Ctrl) in BMSC. **n** No significant change of SOX9 mRNA levels after miR-365 transfection in BMSC. **o** The ACAN mRNA level was significantly decreased by miR-365 transfection in BMSC. **p** No significant change of IHH mRNA levels after miR-365 transfection in BMSC. **q** The RUNX2 mRNA level was significantly increased by miR-365 transfection in BMSC. **r** No significant change of ADAMTS5 mRNA levels after miR-365 transfection in BMSC. **s** No significant change of COL1A1 mRNA levels after miR-365 transfection in BMSC. **t** No significant change of OCN mRNA levels after miR-365 transfection in BMSC. **u** MiR-365 levels were significantly decreased after transfection of miR-365 inhibitor (antagonist) in comparison to control (Inhibitor Ctrl) in OASC2. **v** The SOX9 mRNA level was significantly inhibited by miR-365 inhibitor in OASC2. **w** The ADAMTS5 mRNA level was significantly inhibited by miR-365 inhibitor in OASC2. **x** The RUNX2 mRNA level was significantly inhibited by miR-365 inhibitor in OASC2. **y** The COL1A1 mRNA level was significantly inhibited by miR-365 inhibitor in OASC2. **z** The OCN mRNA level was significantly inhibited by miR-365 inhibitor in OASC2. **aa** MiR-365 levels were significantly decreased after transfection of miR-365 inhibitor (antagonist) in comparison to control (Inhibitor Ctrl) in BMSC. **ab** No significant change of SOX9 mRNA levels after miR-365 inhibitor transfection in BMSC. **ac** No significant change of ADAMTS5 mRNA levels after miR-365 inhibitor transfection in BMSC. **ad** No significant change of RUNX2 mRNA levels after miR-365 inhibitor transfection in BMSC. **ae** The COL1A1 mRNA level was significantly inhibited by miR-365 inhibitor in BMSC. **af** The OCN mRNA level was significantly increased by miR-365 inhibitor in BMSC. Data are presented as mean ± SD from at least 3 sample images for each group; *p ≤ 0.05; **p ≤ 0.01; ****p ≤ 0.0001, relative to indicated control groups respectively

Chondrogenic induction resulted in an increase of the miR-365 levels in both OASC2 and BMSC, although the increase occurred later (after day 7) in OASC2 (Fig. 5a, b). Transfection of miR-365 induced the expression of chondrogenic, hypertrophic, and osteogenic genes including SOX9, ACAN, IHH, RUNX2, ADAMTS5, COL1, and OCN in OASC2 at both mRNA and protein levels (Fig. 5c–l). However, transfection of miR-365 only increased the expression of RUNX2 significantly in BMSC (Fig. 5q). It also inhibited ACAN, a chondrogenic marker in BMSC (Fig. 5o).

### MiR-365 inhibition suppresses the expression of chondrogenic, hypertrophic, and osteogenic markers in OASC2

To determine whether miR-365 is required for the expression of chondrogenic, hypertrophic, and osteogenic genes in OASC2, we suppressed miR-365 expression by transfecting miR-365 antagomir (inhibitor) into OASC2 (Fig. 5u) and BMSC (Fig. 5aa). Inhibition of miR-365 significantly decreased the expression of chondrogenic, hypertrophic, and osteogenic genes including SOX9, ADAMTS5, RUNX2, COL1A1, and OCN in OASC2 (Fig. 5v–z). While in BMSC, only the expression of COL1A1 (Fig. 5ae) was significantly decreased after miR-365 inhibitor treatment.

### MiR-365 increases the MSC and hypertrophic cartilage marker gene expression

**Fig. 6:**
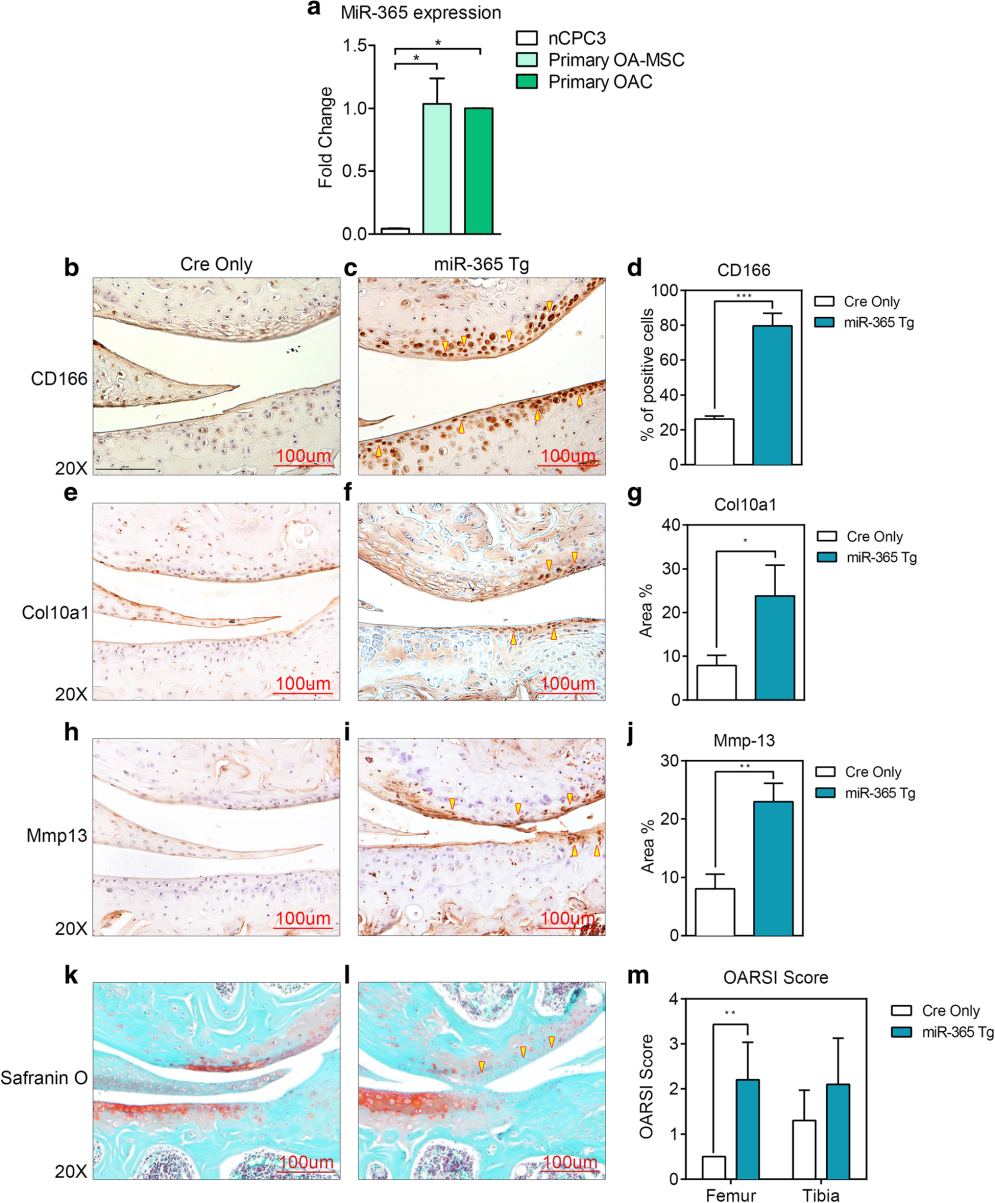
MiR-365 expression in human cartilage cells; and protein expression of MSC marker CD166, hypertrophic cartilage markers Col10a1 and Mmp13 in articular cartilage of miR-365 transgenic mice (Tg). **a** A bar graph showing quantification of miR-365 expression in normal cartilage progenitor cell line 3 (nCPC3), primary OA-MSC, and primary OA chondrocytes (OAC) respectively. Representative immunohistochemistry staining of CD166 (**b**, **c**), Col10a1 (**e**, **f**), and Mmp13 (**h**, **i**) in 6-month-old miR-365 Tg mice (MiR-365 fl +/−; Col2a1-Cre+/−) and age-matched control Cre-only mice (MiR-365 fl −/−; Col2a1-Cre+/−) knee articular cartilage. **d** Quantification of CD166 positive cell numbers in **b** and **c**. **g** Quantification of Col10a1 positive tissue areas in **e** and **f**. **j** Quantification of Mmp13 positive tissue areas in **h** and **i**. **k**, **l** Representative Safranin O staining of 6-month-old miR-365 Tg mice (MiR-365 fl +/−; Col2a1-Cre+/−) and age-matched control Cre-only mice (MiR-365 fl −/−; Col2a1-Cre+/−) knee articular cartilage. **m** OARSI scoring of **k** and **l**. Arrows point to the immuno-positive signals. The images are representative of analyses of at least three mice per group. Data are presented as mean ± SD from at least 3 sample images for each group; *p ≤ 0.05; **p ≤ 0.01; ****p ≤ 0.0001, relative to indicated control groups respectively

To understand the biological significance of miR-365 regulation of cartilage cells, we quantified miR-365 levels in normal human cartilage-derived progenitor cells (nCPC), primary human OA-MSC, and primary human OA chondrocytes. Human OA cartilage cells including both chondrocytes and progenitor stem cells have significantly higher miR-365 levels than normal human cartilage-derived progenitor cells (Fig. 6a). Thus, miR-365 is upregulated in primary OA cartilage cells. To understand the effect of upregulation of miR-365 on cartilage, we performed immunohistochemistry with adult miR-365 transgenic mice (miR-365 fl+/−; Col2a1-Cre+/−) generated in our laboratory [14]. In the miR-365 Tg mice, miR-365 was over-expressed about sixfold in cartilage specifically [14]. It resulted in an increase of hypertrophic markers (Col10a1, Mmp13) and MSC marker (CD166) in cartilage (Fig. 6b–j) and significant proteoglycan loss in the knee articular surface indicated by Safranin O staining (Fig. 6k–m). Furthermore, some of the positive immuno-reactive signals of Col10a1 and Mmp13 occurred in the superficial zone where CD166-positive MSCs resided (arrows, Fig. 6c, f, i). It suggests that the cartilage MSCs may be involved in hypertrophic marker expression.

## Discussion

Mesenchymal stem cells (MSC), a popular source for cell-based cartilage repair, can be derived from a variety of human tissues of the mesenchymal origin including adipose [17], synovium [18], cartilage [19], and bone and bone marrow [20]. Among them, bone marrow-derived MSCs (BMSC) are the most studied and widely used [21]. Despite the success of inducing chondrogenesis using the BMSC, it usually requires long-term pellet culture in 3D [22]. Furthermore, they are prone to hypertrophy, which is undesirable for maintaining hyaline cartilage phenotype [23–25]. These properties contribute to the less-than-ideal long-term success rate of cell-based cartilage repair using the BMSC.

The MSCs in articular cartilage are thought to have inherent advantages for cartilage repair because of their potential roles as chondro-progenitor cells in the cartilage tissue [25]. Furthermore, these MSCs can be isolated from discarded articular cartilage samples of osteoarthritis patients undergoing joint replacement surgery, which have been termed OA-MSC [3]. Using multiple cell lines generated from the OA-MSC including the human OASC2 line used in this study, we showed that OA-MSCs are prone to chondrocyte hypertrophy, which was similar to BMSC [23–25]. However, there is no side-by-side comparison between OA-MSC and BMSC in terms of their differentiation potentials. Furthermore, the gene(s) regulating OA-MSC differentiation is not known. The current study was carried out to gain this knowledge.

### Chondrogenesis

We show that, upon induction with the chondrogenesis medium, the OASC2 cells underwent rapid chondrogenesis in the monolayer cell culture in 7 days. This was reflected by the positive Alcian blue staining and induction of the chondrogenic markers including SOX9, COL2A1, and ACAN. In contrast, human BMSC failed to undergo chondrogenesis under the same condition for 14 days. This was consistent with the previous observation that chondrogenesis of the human BMSC usually requires long-term incubation in a 3D pellet culture after chondrogenic induction [22]. Thus, the OA-MSC cells seemed to have much more enhanced chondrogenic potentials than BMSCs.

### Mineralization, hypertrophy, and osteogenesis

To our surprise, we observed potent and rapid mineralization of human OA-MSC cells upon chondrogenic induction. Concurrent with chondrogenesis, mineralization of the OA-MSC cells, as indicated by the strong positive Alizarin red staining, occurred in 7 days after chondrogenic induction. In contrast, the BMSC cells only exhibited minor residual Alizarin red staining in 14 days after induction. Notably, such strong mineralization in the OA-MSC occurred after chondrogenic induction, which normally does not activate the cell mineralization process. To determine whether mineralization of OA-MSC was due to chondrocyte hypertrophy or osteogenesis of the stem cells, we quantified the marker genes of hypertrophy and osteogenesis respectively. The results indicated that both markers of chondrocyte hypertrophy and stem cell osteogenesis were induced by the chondrogenesis medium in the OA-MSC. Thus, both the hypertrophy and osteogenesis differentiation processes may account for mineralization of the OA-MSC. In contrast, only hypertrophy-related genes such as COL10A1 and RUNX2 were activated by the chondrogenic induction in the BMSC, consistent with the previous observation that the BMSC was prone to hypertrophy during chondrogenesis. However, the induction of hypertrophy was not sufficient to induce mineralization in the BMSC.

We have shown previously that, between the two groups of human OA-MSC lines tested, the OASC2 group had stronger chondrogenesis potential but less osteogenesis potential than the OASC18 group [3]. However, the osteogenesis potentials were only tested during the osteogenic induction by the osteogenesis medium. Thus, it is a novel finding that the human OA-MSC undergoes rapid mineralization during chondrogenesis. It is not a sequential activation of chondrogenesis followed by hypertrophy and osteogenesis, as occurred in the osteochondro-progenitor cells during endochondral ossification of skeletal development [26]. Rather, it seemed to be a concurrent event with chondrogenesis, hypertrophy, osteogenesis, and mineralization occurring simultaneously. This conclusion was supported by the observation that both chondrogenesis, as reflected by Alcian blue staining, and mineralization, as reflected by Alizarin red staining, occurred simultaneously by day 7 and further enhanced by day 14. In addition, the gene expression of the markers of chondrogenesis, hypertrophy, and osteogenesis was upregulated concurrently rather than sequentially. This was shown not only at the mRNA levels by real-time quantitative RT-PCR, but also at the protein levels by western blot analysis. We hypothesize that such concurrent differentiation in the OA-MSC cells may be a specific property of the MSCs in the adult cartilage tissue. Alternatively, it can also be a unique property of the MSCs during OA pathogenesis. Indeed, the hallmarks of OA include activation of the hypertrophic genes such as type X collagen and transcriptional factor Runx2, enhanced mineralization [27], and abnormal induction of osteogenesis [28], all of which occur in the OA-MSC. It may also suggest that some of the pathological features of OA may be derived from the OA-MSC. Thus, it is highly important to understand the molecular mechanisms that regulate OA-MSC differentiation processes.

### A master gene that regulates OA-MSC differentiation

Through a candidate screening approach, we identified miR-365 as a master gene that induced gene expression of the markers of chondrogenesis, hypertrophy, and osteogenesis in the OA-MSC cells. Since OA-MSCs were derived from human OA cartilage and might participate in OA pathogenesis, we focused on the molecules that were activated by mechanical loading and inflammation, the two main factors involved in the OA onset and progression [29, 30]. MiR-365 is one of the first mechanical-sensitive microRNAs (mechanomiR) identified in cartilage, which is involved in the mechanical activation of chondrocyte differentiation [12]. It is also activated by IL-1 [13], a major inflammatory cytokine in OA, and involved in IL-6 regulation [31].

In order for a master gene to regulate multiple differentiation processes, it has to regulate multiple targets. MicroRNA is well suited for this task because it targets the 3′ UTR of multiple RNAs in a variety of cells. For example, miR-365 activates chondrogenesis and chondrocyte hypertrophy by inhibiting histone deacetylase 4 (HDAC4), a potent inhibitor of chondrocyte hypertrophy [12]. The activation of hypertrophy is also achieved by activation of the hedgehog pathway, a major signaling pathway required for OA pathogenesis [32]. During development, miR-365 is expressed by per-hypertrophic chondrocytes, coinciding with Indian hedgehog (Ihh) expression [12]. On the other hand, miR-365 also promotes osteogenesis through targeting HDAC4 in pre-osteoblasts MC-3 T3 [33]. Thus, miR-365 could promote chondrogenesis, hypertrophy, and osteogenesis in different types of cells. However, it was not known whether it did so in the OA-MSC and BMSC.

Our results indicated that miR-365 was both necessary and sufficient for activation of the marker genes for chondrogenesis, hypertrophy, and osteogenesis in the OA-MSC. Transfection of the miR-365 mimic activated the gene expression of chondrogenesis (SOX9, ACAN), hypertrophy (IHH, ADAMTS5), and osteogenesis (RUNX2, COL1A1, and OCN), while transfection of miR-365 antagomiR inhibited the mRNA levels of these genes. However, in the BMSC cells, such concurrent differentiation processes did not occur and miR-365 appeared to inhibit chondrogenesis. Thus, the OA-MSC activity, as reflected by the concurrent differentiation, can be induced by miR-365. Conversely, the activity of the OA-MSC can be inhibited by the miR-365 inhibitor.

It is not known whether the concurrent differentiation of chondrogenesis, hypertrophy, and osteogenesis occurred in the same or different OA-MSC cells. Since the OASC2 cell line was derived from a single OA-MSC in adult human cartilage, it strongly suggested that the concurrent differentiation events occur simultaneously in the same cells. However, a possibility still existed that different cells from the same cell population might differentiate towards different cell lineages. Such possibility requires a single-cell analysis tracking the expression of multiple genes in one cell. To eliminate the heterogeneity of primary OA-MSCs, especially regarding the concurrent differentiation events, we performed the analyses using the OASC2 cell line. We validated the elevated miR-365 levels in primary OA-MSC compared to normal cartilage-derived MSC. We demonstrated that the cartilage-specific expression of miR-365 increased OA-MSC and hypertrophic markers expression in transgenic mice in vivo. Furthermore, some of the hypertrophic markers were co-localized with OA-MSC in articular cartilage. This is consistent with the previous observation that the percentage of cartilage MSCs increase during OA pathogenesis [4, 12].

In conclusion, our data for the first time show that induction of chondrogenesis can promote chondrogenic, hypertrophic and osteogenic genes in the OA-MSC and this regulation can be mediated by miR-365. In comparison to the BMSC, which are widely used for cell-based cartilage repair, OA-MSCs can undergo rapid chondrogenesis in monolayer cell culture in 7 days while the BMSC chondrogenesis requires long-term cell culture in 3D. Chondrogenic induction also activated mineralization in the OA-MSC but not in the BMSC, while both types of cells underwent hypertrophy. MiR-365 expression activated chondrogenic, hypetrophic, and osteogenic gene expression in OA-MSC in vitro and OA pathogenesis in vivo. These findings have important implications for cell-based cartilage repair using either OA-MSC or BMSC cells and present a new paradigm in which chondrogenesis, mineralization, and the expression of OA related genes may be coupled during the OA-MSC differentiation process. Our data suggests that miR-365, a potential master regulator of the OA-MSC, can be used to modulate the OA-MSC activities during cartilage repair.

## Additional files

Additional file 1: **Figure S1.** Western blot densitometric analysis of chondrogenic markers, hypertrophic markers, and osteogenic markers after chondrogenic induction in OASC2. To quantify the protein expression level of SOX9 (a), RUNX2 (b), COL10A1 (c), and OCN (d) in OASC2 which underwent chondrogenic induction, densitometric analysis of the western blots in terms of gray intensity was performed using ImageJ for at least three experimental repeats. The densitometric intensity was normalized to that of β-ACTIN which served as the loading control. The average of the Day3-Growth group was normalized to 1 fold. Data are presented as mean ± SD for each group; **p < 0.01, ***p < 0.001, ****p < 0.0001, *******p < 0.0000001. (JPG 1473 kb)

Additional file 2: **Figure S2.** Western blot densitometric analysis of chondrogenic markers and hypertrophic markers in undifferentiated OASC2 upon transfection of miR-365 or its inhibitor. To quantify the protein expression level of MMP13 (a), COL10A1 (b), ADAMTS-5 (c), SOX9 (d) and RUNX2 (e) in undifferentiated OASC2 transfected with miR-365 mimic or miR-365 inhibitor, densitometric analysis of the western blots in terms of gray intensity was performed using ImageJ for at least three experimental repeats. The densitometric intensity was normalized to that of β-ACTIN which served as the loading control. The average of the mimic control group was normalized to 1 fold. Data are presented as mean ± SD for each group; *p < 0.05, **p < 0.01, ***p < 0.001, ****p < 0.0001, *****p < 0.00001, *********p < 0.000000001. (JPG 1504 kb)

## Data Availability

The datasets used and/or analyzed during the current study are available from the corresponding author on reasonable request.
