# Supplementary figures and images for: Chondrogenic induction of human osteoarthritic cartilage-derived mesenchymal stem cells activates mineralization and hypertrophic and osteogenic gene expression through a mechanomiR
Source: Arthritis Res Ther. 2019 Jul 8;21:167. doi: 10.1186/s13075-019-1949-0 (PMC6615283; doi:10.1186/s13075-019-1949-0)

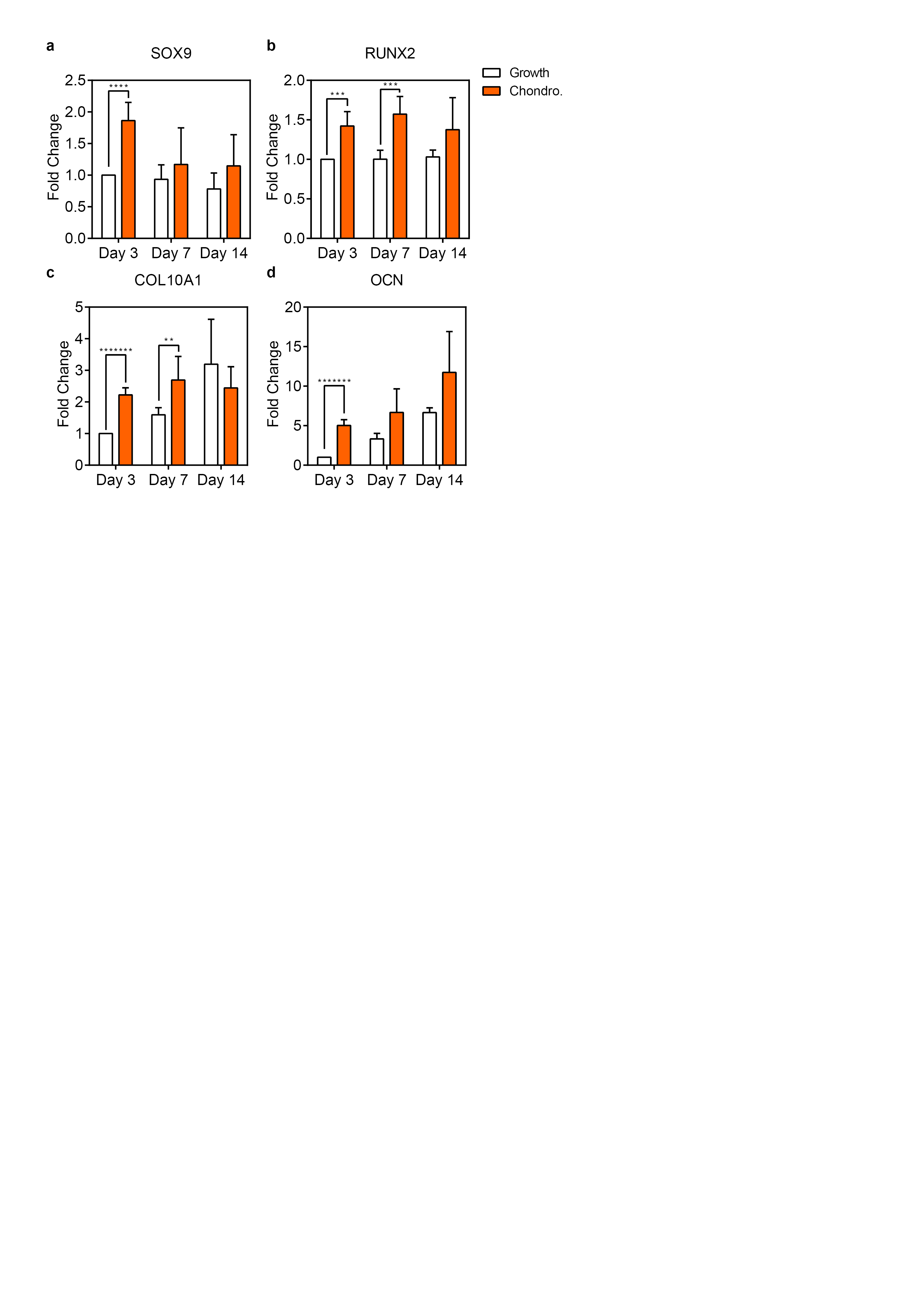

Supplement: Supplementary file 1 — Figure S1. Western blot densitometric analysis of chondrogenic markers, hypertrophic markers, and osteogenic markers after chondrogenic induction in OASC2. To quantify the protein expression level of SOX9 (a), RUNX2 (b), COL10A1 (c), and OCN (d) in OASC2 which underwent chondrogenic induction, densitometric analysis of the western blots in terms of gray intensity was performed using ImageJ for at least three experimental repeats. The densitometric intensity was normalized to that of β-ACTIN which served as the loading control. The average of the Day3-Growth group was normalized to 1 fold. Data are presented as mean ± SD for each group; **p < 0.01, ***p < 0.001, ****p < 0.0001, *******p < 0.0000001. (JPG 1473 kb) [file 13075_2019_1949_MOESM1_ESM.jpg]

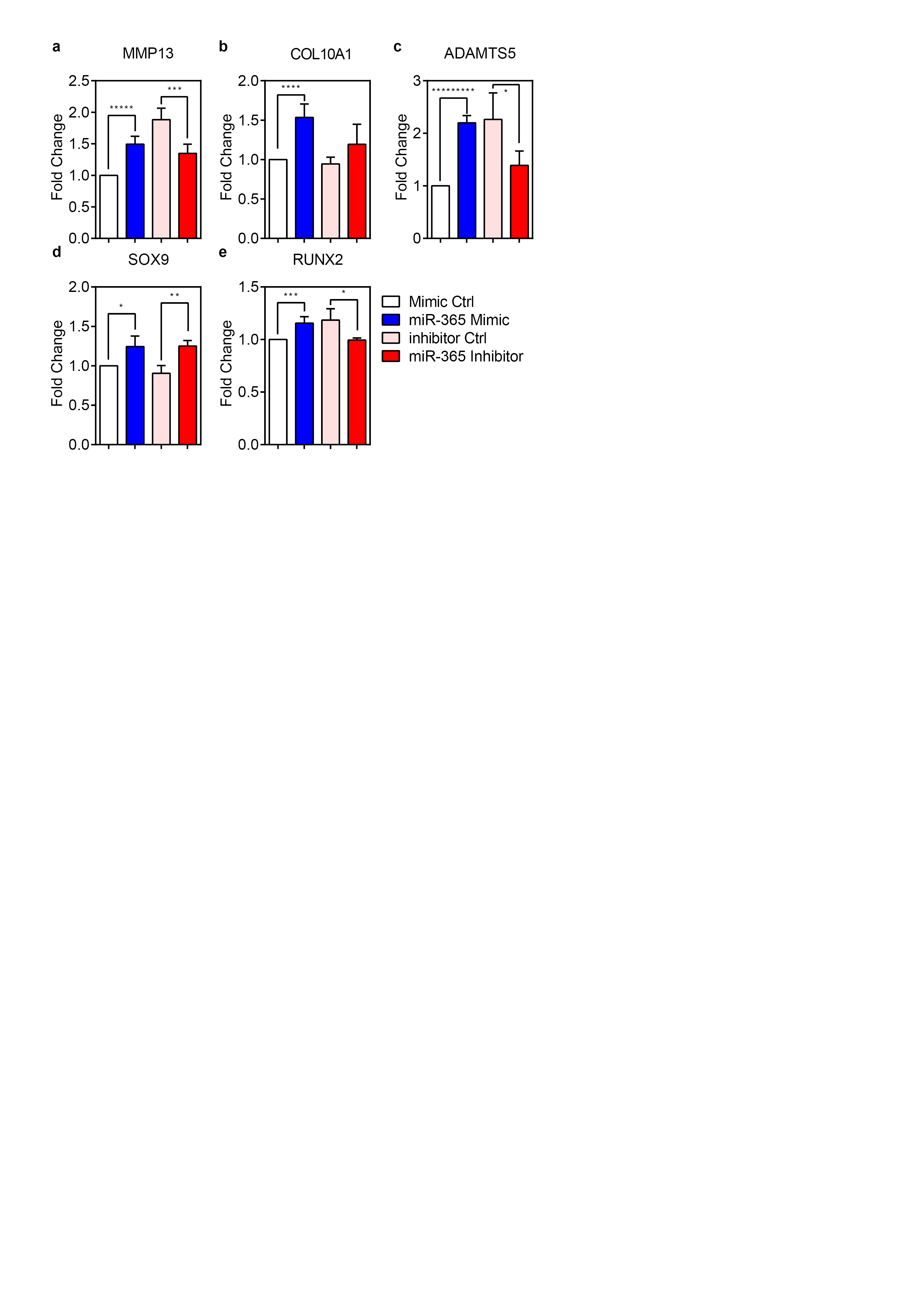

Supplement: Supplementary file 2 — Figure S2. Western blot densitometric analysis of chondrogenic markers and hypertrophic markers in undifferentiated OASC2 upon transfection of miR-365 or its inhibitor. To quantify the protein expression level of MMP13 (a), COL10A1 (b), ADAMTS-5 (c), SOX9 (d) and RUNX2 (e) in undifferentiated OASC2 transfected with miR-365 mimic or miR-365 inhibitor, densitometric analysis of the western blots in terms of gray intensity was performed using ImageJ for at least three experimental repeats. The densitometric intensity was normalized to that of β-ACTIN which served as the loading control. The average of the mimic control group was normalized to 1 fold. Data are presented as mean ± SD for each group; *p < 0.05, **p < 0.01, ***p < 0.001, ****p < 0.0001, *****p < 0.00001, *********p < 0.000000001. (JPG 1504 kb) [file 13075_2019_1949_MOESM2_ESM.jpg]
